# CHRNA5/CHRNA3 gene cluster is a risk factor for lumbar disc herniation: a case-control study

**DOI:** 10.1186/s13018-019-1254-2

**Published:** 2019-07-30

**Authors:** Xuejun Yang, Xiaodong Guo, Zhi Huang, Yifeng Da, Wenhua Xing, Feng Li, Manglai Li, Ke Sun, Haiyu Jia, Yong Zhu

**Affiliations:** 1 Spine (Thoracic and Vertebra) Department, the Second Affiliated Hospital of Inner Mongolia University, #1 Yingfang Road, Hohhot, 010050 Inner Mongolia China; 2 Inner Mongolia People’s Hospital, Hohhot, Inner Mongolia China; 3 Inner Mongolia Medical University, Hohhot, Inner Mongolia China; 4 The Affiliated Hospital of Inner Mongolia Medical College, #1 North Tongdao Road, Hohhot, 010020 Inner Mongolia China

**Keywords:** Lumbar disc herniation, *CHRNA5*/*CHRNA3*, Susceptibility, Single-nucleotide polymorphism

## Abstract

**Background:**

Lumbar disc herniation, a type of chronic low back pain syndrome, is caused by the lumbar intervertebral disk degeneration. Genetic variation in the *CHRNA5*/*CHRNA3* has shown strong associations with smoking-related diseases. This study’s aim is to test whether single-nucleotide polymorphisms in the *CHRNA5*/*CHRNA3* gene are associated with lumbar disc herniation risk.

**Methods:**

The genotype frequency distributions of the polymorphisms were detected by polymerase chain reaction-restriction fragment length polymorphism in 380 lumbar disc herniation patients (case group) and 400 healthy individuals (control group). Allelic, genotypic, and haplotype analyses were performed.

**Results:**

We found that the individuals with rs8040868 CT genotype had a 0.46-fold higher risk of lumbar disc herniation than those with rs8040868 TT genotype, in men group (OR = 0.46, 95% CI 0.25–0.84, *p* = 0.012). Also among women, rs8040868 CT + CC genotype still reduced the risk of lumbar disc herniation under the dominant model (OR = 0.50, 95% CI 0.28–0.89, *p* = 0.019). Haplotype analysis showed that compared with the *CHRNA5* “TACAACCG” wild-type, the “TACACCCG” haplotype was found to be associated with a decreased risk of lumbar disc herniation (LDH) (OR = 0.79, 95% CI 0.63–1.00, *p* = 0.047), while, in the less than 50-year-old group, CHRNA5 “TACACCCG” increased the risk of LDH (OR = 1.46, 95% CI 1.01–2.13, *p* = 0.047).

**Conclusions:**

Our data suggest that gene variance in the *CHRNA5*/*CHRNA3* is associated with risk of lumbar disc herniation in the case-control study.

## Introduction

Lumbar disc herniation (LDH) is one of the more common spinal diseases caused by the degeneration and the displacement of nucleus pulposus or annulus fibrosis beyond the intervertebral disc space [1]. LDH is characterized with low back and leg pain resulting from the degenerated lumbar disc compressing the spinal nerve root [2]. Many studies have demonstrated that 70–85% of all people will suffer from low back pain at some time in life [3]. As LDH is considered a significant health care problem involving multifactorial interactions, numerous studies have been performed to identify the risk factors. Previous etiologic studies have focused on environmental risk factors, such as sex, age, height, smoking habits, and occupational factors [4–6].

As we all know, smoking is associated with increased morbidity, mortality, and personal and public cost. An early study demonstrated increased vertebral bone porosity and reduced trabecular bone thickness in mice chronically exposed to tobacco smoke [7]. Likewise, according to Nasto LA et al. [8], short-term exposure to high levels of primary tobacco smoke inhalation promotes degeneration of vertebral bone and discs, and then clearly established a direct cause and effect relationship between smoking and spine degeneration in mice. Moreover, the findings also suggested that smoking adversely affects spine health only in part through DNA damage. However, not all smokers develop LDH, a fact that might indicate that genetic variability also may play a significant role in the pathogenesis of LDH [9–11]. Genetic predisposition has been widely acknowledged in LDH. Several genes such as *COL1A1* [12], *MMPs* [13], *COL9A2* [14], and *ADAMTS*-*5* [15] have been reported to be associated with LDH. Cigarette smoking is a genetically influenced addictive behavior [16, 17], and nicotine is the main component of cigarette smoke responsible for smoking dependence [18]. Several studies have indicated that the cluster of cholinergic receptor nicotinic α genes on chromosome 15q25, encoding the alpha5 and alpha3 subunits of the nicotinic acetylcholine receptors (*CHRNA5* and *CHRNA3*), are significantly related to smoking behavior [19, 20]. However, there is no investigation that found any association between genetic variations in the nicotinic acetylcholine receptor (nAChR) gene cluster *CHRNA5*/*CHRNA3* and LDH. This is, to our knowledge, the first case-control study that investigated the association between *CHRNA5*/*CHRNA3* gene polymorphisms and LDH in a Chinese population with LDH.

## Material and methods

### Subjects

For this case-control association analysis of LDH, a total of 380 LDH patients and 400 unrelated healthy controls were recruited from Xi’an Honghui hospital. All participants lived in Xi’an area, and all participants were Han people. So, there are no regional and ethnic differences. The diagnosis of LDH required the following criteria: (1) patients who had a history of lumbar sprain and/or a history of chronic strain, (2) patients who had pain in the inferior lumbar part of the spine and regional sciatic nerve pain in the leg caused by bed rest, (3) patients with tenderness beside the lumbar spine that affects the leg or foot, (4) patients whose lumbar flexion range was obviously limited, (5) patients with positive results in the straight-leg raising test and augmentation test (Bragard’s sign), and (6) patients who had the following nerve injury symptoms: muscular atrophy, motor weakness, decreased sensation, and hyporeflexia. Meeting any one of (1)–(6) and then combined with patients with clinical manifestations of LDH in accordance with imaging findings, including computed radiography, computed tomography, and/or magnetic resonance imaging, is considered positive for LDH. Primary exclusion criteria included spinal and joint diseases such as trauma, spinal tumor, synovial cyst, inflammatory disease, scoliosis, osteoarthritis, spondylosis, and spondylolisthesis. Moreover, patients who have history of labor work or heavy smoking were also excluded. According to the Labor Protection Measures Standard for Heavy Labor Work (No. 103007971) and the National Standard “Classification of Physical Labor Intensity” (No. BG386983), we exclude labor worker by inquiring about the nature of their work and the intensity of their physical consumption. By referring to the literature [21], according to the smoking index is equal to the number of cigarettes per day multiplied by the number of years of smoking (365 day/year), the smoking index ≥ 60 pack years is defined as a heavy smoker, using this criterion to distinguish whether the patient is a severe smoker. Four hundred unrelated healthy controls were recruited from Xi’an Honghui hospital. Inclusion criteria of the control group were (1) good health as confirmed by physical examination, (2) no recent infections, (3) no history of tumors, and (4) history of lumbar sprain and/or chronic strain.

Ahsan et al. adopted a retrospective case-control study found that physical exertion, work stress, and daily work time of more than 8 h were highly correlated with the occurrence of LDH [22]. An HS and other studies found that smokers had a 50% increased risk of disc herniation compared with non-smokers [23]. It shows that heavy labor or smoking has a greater impact on lumbar disc herniation. So, we exclude labor worker or heavy smoker.

### Genotyping of SNPs

Fifteen tag single-nucleotide polymorphisms (SNPs) of CHRNA5 and CHRNA3 were selected from the NCBI SNP database (www.ncbi.nlm.nih.gov/SNP) and the HapMap database (www.hapmap.org). All the SNPs selected had a minor allele frequency (MAF) of more than 0.05. And primers for 15 tag SNP typing were designed by Agena on-line design software (https://agenacx.com/online-tools/).

Genomic DNA was extracted from peripheral blood leukocytes of affected individuals and controls using standard protocols. The DNA quantity was evaluated by spectrometry (DU530UV/VIS spectrophotometer, Beckman Instruments, Fullerton, CA, USA). The SNP genotyping was performed on the Agena MassARRAY SNP genotyping platform (Agena Bioscience, San Diego, CA, USA) according to the manufacturer’s protocol.

### Statistical analyses

Statistical analysis was performed using SPSS version 21.0 (SPSS Inc., Chicago, IL, USA). Hardy–Weinberg equilibrium was assessed by using a chi-square test. Measurement data were expressed as the mean ± standard deviation (SD). The difference between the two groups was compared using a *t* test. In a multivariate logistic regression model, we assessed the independent association between CHRNA3/CHRNA5 gene polymorphism, smoking, drinking, and risk of LDH. Odds ratios (ORs) and 95% confidence intervals (CIs) were calculated to determine the relative risk of LDH. Haplotype blocks and the linkage disequilibrium (LD) patterns were estimated by using the Haploview program (version 4.2, Broad Institute of MIT, and Harvard, Cambridge, MA, USA). Linkage disequilibrium coefficients (*D*’ and *r*^2^) were calculated as described previously [24]. All tests were two-sided, and *p* < 0.05 indicated a significant difference.

## Results

### Study population

The general characteristics of both groups are summarized in Table 1. A total of 780 Northwest Chinese subjects with Han ethnicity were recruited in this case-control study, comprising 380 patients with LDH (152 females and 228 males; mean age 50.43 ± 12.27 years) and 400 healthy controls (166 females and 134 males; mean age 50.79 ± 8.13 years). No significant differences in gender and age were found between the two groups. Of the 380 patients, 43 were smokers and 337 were non-smokers; 12 were drinking and 368 were non-drinking.

**Table 1:** The general characteristics of study subjects

Independent variables	LDH patients	Healthy controls	*p* value
	(*n* = 380)	(*n* = 400)	
Age			
≤ 50 [*N* (%)]	177 (46.6%)	203 (51.0%)	
> 50 [*N* (%)]	203 (53.4%)	197 (49.0%)	
Gender			0.610
Female [*N* (%)]	152 (40%)	166 (41.0%)	
Male [*N* (%)]	228 (60%)	234 (59.0%)	
Mean age (mean ± SD, years)	50.43 ± 12.27	50.79 ± 8.13	0.629
Smoking			
Yes	43 (11.3%)	186 (46.5%)	
No	337 (88.7%)	214 (53.5%)	
Drinking			
Yes	12 (3.2%)	168 (42.0%)	
No	368 (96.8%)	232 (58.0%)	

*LDH* lumbar disc herniation

*p* < 0.05 indicates statistical significance

### Hardy–Weinberg equilibrium and SNP alleles

The basic information about all the SNPs including gene, band, position, alleles, and Hardy–Weinberg equilibrium (HWE) results are presented in Table 2. The results of the HWE showed that the genotype frequency distributions of *CHRNA5*/*CHRNA3* in the case and control groups were in line with genetic balance (all *p* > 0.05), which showed that all of the 15 tag SNPs were at equilibrium and were representative. In the allele model, we found that there was no site affected by the genetic risk of lumbar disc herniation. Genetic models (genotype, dominant, recessive, and additive) and the genotype frequencies were used to further identify the associations between the SNPs and the risk of LDH. The results without stratification showed no association between SNPs and the risk of LDH (data were not shown).

**Table 2:** Allele frequencies in cases and controls and OR estimates for LDH

SNP ID	Gene	Alleles A/B	Case			Control			MAF		HWE, *p* †	Allele model	
			AA count	AB count	BB count	AA count	AB count	BB count	Case	Control		OR (95% CI)	*p* ‡
rs667282	CHRNA5	C/T	90	187	102	91	196	113	0.484	0.473	0.764	1.05 (0.86–1.28)	0.645
rs16969948	CHRNA5	G/A	1	39	339	2	42	356	0.054	0.058	0.377	0.94 (0.61–1.45)	0.770
rs588765	CHRNA5	T/C	15	133	232	17	121	262	0.214	0.194	0.523	1.14 (0.89–1.45)	0.310
rs6495306	CHRNA5	G/A	15	132	232	17	121	262	0.214	0.194	0.523	1.13 (0.88–1.45)	0.328
rs17486278	CHRNA5	C/A	27	133	219	29	163	208	0.247	0.276	0.803	0.86 (0.68–1.08)	0.185
rs680244	CHRNA5	T/C	27	151	202	26	149	224	0.270	0.252	0.894	1.10 (0.87–1.38)	0.422
rs569207	CHRNA5	T/C	94	185	100	91	196	113	0.492	0.473	0.764	1.08 (0.89–1.32)	0.439
rs692780	CHRNA5	C/G	18	131	231	19	124	257	0.220	0.203	0.438	1.11 (0.87–1.42)	0.404
rs3743077	CHRNA3	T/C	21	140	219	21	136	243	0.239	0.223	0.772	1.10 (0.87–1.39)	0.426
rs1317286	CHRNA3	G/A	5	61	314	6	64	330	0.093	0.095	0.150	0.98 (0.70–1.38)	0.915
rs938682	CHRNA3	G/A	87	179	114	78	196	125	0.464	0.441	1.000	1.10 (0.90–1.34)	0.354
rs12914385	CHRNA3	T/C	25	136	219	26	165	209	0.245	0.271	0.448	0.87 (0.69–1.09)	0.232
rs2869546	CHRNA3	C/T	19	139	222	20	135	242	0.233	0.220	0.884	1.07 (0.85–1.36)	0.557
rs3743075	CHRNA3	T/C	71	183	117	92	181	123	0.438	0.461	0.107	0.91 (0.75–1.12)	0.369
rs8040868	CHRNA3	C/T	36	157	186	40	186	174	0.302	0.333	0.369	0.87 (0.70–1.08)	0.198

*LDH* lumbar disc herniation, *SNP* single-nucleotide polymorphism, *MAF* minor allele frequency, *HWE* Hardy–Weinberg equilibrium, *OR* odds ratio, *95*% *CI* 95% confidence interval

† *p* was calculated by exact test

‡ *p* was calculated by Pearson chi-squared test

### Association between *CHRNA5*/*CHRNA3* and the risk of LDH

In a multivariate logistic regression model, we assessed the independent association between CHRNA3/CHRNA5 gene polymorphism, smoking, drinking, and risk of LDH. We found that the individuals with rs8040868 CT genotype had a 0.46-fold higher risk of lumbar disc herniation than those with rs8040868 TT genotype, in the men group (OR = 0.46, 95% CI 0.25–0.84, *p* = 0.012) (Table 3). Also among women, rs8040868 CT + CC genotype still reduced the risk of lumbar disc herniation under the dominant model (OR = 0.50, 95% CI 0.28–0.89, *p* = 0.019) (Table 3).

**Table 3:** Stratified analysis of rs8040868 polymorphisms of CHRNA3 gene by gender and risk of LDH

Group	Variants	OR (95%CI)	*p*	Group	Variants	OR (95%CI)	*p*
≤ 50	Age	0.91 (0.87–0.96)	< 0.001	> 50	Age	1.07 (1.02–1.11)	0.004
	Smoking	0.17 (0.07–0.43)	< 0.001		Smoking	0.11 (0.06–0.24)	< 0.001
	Drinking	0.05 (0.02–0.15)	< 0.001		Drinking	0.07 (0.03–0.18)	< 0.001
	Gender	0.07 (0.03–0.13)	< 0.001		Gender	0.52 (0.29–0.94)	0.032
	rs8040868-TT	1			rs8040868-TT	1	
	rs8040868-CT	1.11 (0.44–2.79)	0.819		rs8040868-CT	1.02 (0.43–2.41)	0.96
	rs8040868-CC	0.64 (0.25–1.64)	0.351		rs8040868-CC	1.07 (0.45–2.5)	0.883
	Age	0.91 (0.87–0.96)	< 0.001		Age	1.07 (1.02–1.11)	0.004
	Smoking	0.17 (0.07–0.42)	< 0.001		Smoking	0.11 (0.06–0.24)	< 0.001
	Drinking	0.05 (0.02–0.15)	< 0.001		Drinking	0.07 (0.03–0.18)	< 0.001
	Gender	0.07 (0.03–0.13)	< 0.001		Gender	0.52 (0.29–0.95)	0.032
	rs8040868-TT VS. (CT + CC)	0.63 (0.37–1.07)	0.084		rs8040868-TT VS. (CT + CC)	1.03(0.62–1.71)	0.909
	Age	0.91 (0.87–0.96)	< 0.001		Age	1.07 (1.02–1.11)	0.004
	Smoking	0.17 (0.07–0.42)	< 0.001		Smoking	0.11 (0.06–0.24)	< 0.001
	Drinking	0.05 (0.02–0.16)	< 0.001		Drinking	0.07 (0.03–0.18)	< 0.001
	Gender	0.07 (0.03–0.13)	< 0.001		Gender	0.52 (0.29–0.94)	0.032
	rs8040868-(TT + CT)VS.CC	1.16(0.48–2.8)	0.749		rs8040868-(TT + CT)VS.CC	0.96 (0.43–2.16)	0.916
Male	Age	0.94 (0.91–0.97)	< 0.001	Female	Age	1.08 (1.05–1.11)	< 0.001
	Smoking	0.12 (0.06–0.21)	< 0.001		Smoking	/	0.999
	Drinking	0.05 (0.03–0.11)	< 0.001		Drinking	/	0.999
	rs8040868-TT	1			rs8040868-TT	1	0.769
	rs8040868-CT	0.46 (0.25–0.84)	0.012		rs8040868-CT	1.12 (0.68–1.85)	0.649
	rs8040868-CC	0.75 (0.28–2.03)	0.572		rs8040868-CC	1.32 (0.59–2.98)	0.502
	Age	0.94( 0.92–0.97)	< 0.001		Age	0.94 (0.92–0.97)	< 0.001
	Smoking	0.12 (0.06–0.21)	< 0.001		Smoking	0.12 (0.06–0.21)	< 0.001
	Drinking	0.06 (0.03–0.11)	< 0.001		Drinking	0.06 (0.03–0.11)	< 0.001
	rs8040868-TT VS. (CT + CC)	0.5(0.28–0.89)	0.019		rs8040868-TT VS. (CT + CC)	0.50 (0.28–0.89)	0.019
	Age	0.94 (0.91–0.97)	< 0.001		Age	1.08 (1.05–1.11)	< 0.001
	Smoking	0.11 (0.06–0.2)	< 0.001		Smoking	/	0.999
	Drinking	0.06 (0.03–0.12)	< 0.001		Drinking	/	0.999
	rs8040868-(TT + CT)VS.CC	1.11 (0.44–2.85)	0.821		rs8040868-(TT + CT)VS.CC	1.25 (0.57–2.72)	0.573

### Association of *CHRNA5* haplotypes with the risk of steroid-induced ONFH

**Fig. 1:**
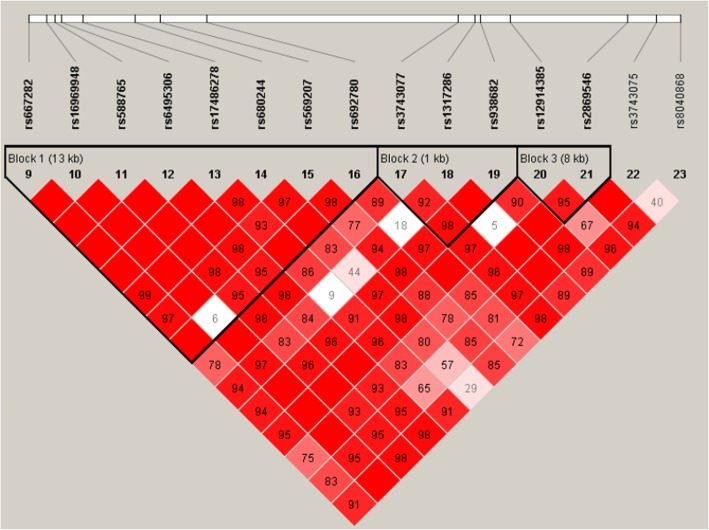
LD structures of the 15 SNPs genotyped in CHRNA5/CHRNA3 gene. The LD strengths between paired SNPs are shown in color according to the confidence intervals’ model. Red squares display statistically significant associations between a pair of SNPs, as measured by *D*’; darker shades of red indicate higher *D*’

Haplotypes were constructed on the basis of the genotype data from 15 SNPs using Haploview software (version 4.2). Linkage disequilibrium *D*’ value between SNPs and the reconstructed LD plots of the 15 SNPs are shown in Fig. 1. Two LD blocks were observed according to the confidence interval method [25] (*D*’ > 0.9 and *r*^2^ > 0.8). The block contained rs667282, rs16969948, rs588765, rs6495306, rs17486278, rs680244, rs569207, and rs6927800; another includes rs3743077, rs1317286, and rs938682. Another block includes rs3743077, rs1317286, and rs938682. Compared with the *CHRNA5* “TACAACCG” wild-type, the “TACACCCG” haplotype was found to be associated with a decreased risk of LDH (OR = 0.79, 95% CI 0.63–1.00, *p* = 0.047; Table 4), while, in the less than 50-year-old group, *CHRNA5* “TACACCCG” increased the risk of LDH (OR = 1.46, 95% CI 1.01–2.13, *p* = 0.047; Table 4).

**Table 4:** Association between haplotype of CHRNA5 regions and LDH in individuals

Analysis	Gene	SNP	Haplotype	OR (95% CI)	*p* value †
Overall	CHRNA5	rs667282|rs16969948|rs588765|rs6495306|rs17486278|rs680244|rs569207|rs692780	TACAACCG	1	
			CACAACTG	1.02 (0.83–1.24)	0.857
			TACACCCG	0.79 (0.63–1.00)	0.047
			TGCAATCG	1.02 (0.60–1.72)	0.943
			TATGATCC	0.90 (0.70–1.15)	0.387
			TGCAATCC	0.94 (0.39–2.29)	0.892
≤ 50	CHRNA5	rs667282|rs16969948|rs588765|rs6495306|rs17486278|rs680244|rs569207|rs692780	TACAACCG	1	
			CACAACTG	1.15 (0.84–1.59)	0.390
			TGCAATCG	0.56 (0.22–1.44)	0.231
			TACACCCG	1.46 (1.01–2.13)	0.047
			TATGATCC	0.84 (0.56–1.25)	0.383
			TGCAATCC	0.9 (0.27–3.02)	0.860

*LDH* lumbar disc herniation

† *p* values were calculated by unconditional logistic regression adjusted for age and gender

## Discussion

In the current study, we examined the genetic associations and interactions between variations in the *CHRNA5*/*CHRNA3* gene cluster and LDH in northern Chinese. Association analyses revealed that *CHRNA3* rs8040868 TC-CC decreased the risk of LDH in the female group and male group. Haplotype analysis revealed that “TACACCCG” haplotype was associated with a decreased risk of LDH, while in the less than 50-year-olds, *CHRNA5* “TACACCCG” increased the risk of LDH. These results suggested that *CHRNA5*/*CHRNA3* gene polymorphisms might be used as genetic determinants for LDH susceptibility.

*CHRNA3* and *CHRNA5*, coding for α3 and α5 subunits of the neuronal nicotinic acetylcholine receptor, have been reported to partially overlap in a tail-to-tail configuration, sharing their 3′ ends in human [26] and bovine genomes [27]. Many studies have demonstrated that *CHRNA3* and *CHRNA5* are functionally linked not only because they code for different subunits of nAChR but also because their protein products can oligomerize to form functional channels, thus suggesting multiple possibilities of reciprocal regulation [28]. Both the *CHRNA3* and *CHRNA5* genes are expressed in human brain regions relevant to nicotine addiction, such as the nucleus accumbens, amygdala, and entorhinal cortex [29]. While many *CHRNA5*/*CHRNA3* gene polymorphisms and linkage studies have been published for numerous smoking-related diseases [30–32], no studies have been performed to indicate relationships between *CHRNA5*/*CHRNA3* and LDH. However, it has been reported that smoking is a causal environmental risk factor for LDH [33], and α5/α3 nicotinic receptor subunit alleles increase risk for heavy smoking [29, 34]. It may be hypothesized that genetic changes in *CHRNA5*/*CHRNA3* could affect the risk of LDH in smokers. We found that the individuals with rs8040868 CT genotype had a 0.46-fold higher risk of lumbar disc herniation than those with rs8040868 TT genotype, in the men group (OR = 0.46). Also among women, rs8040868 CT + CC genotype still reduced the risk of lumbar disc herniation under the dominant model (OR = 0.50). Haplotype analysis revealed that *CHRNA5* “TACACCCG” haplotype was associated with a decreased risk of LDH in overall analysis, while in the less than 50-year-olds, *CHRNA5* “TACACCCG” increased the risk of LDH. The stratified analysis uses a small sample size, which may cause false positive results. Then whether the haplotype reduced or increased the risk, we still need a large sample to verify, while eliminating as much as possible the impact of other confounding factors on the disease.

These results suggested that *CHRNA5*/*CHRNA3* gene polymorphisms might be used as genetic determinants for LDH susceptibility. The studies have reported associations between rs8040868 and lung cancer risk [35] and schizophrenia risk [36]. As predicted by HaploReg v4.1 (https://pubs.broadinstitute.org/mammals/haploreg/haploreg.php), rs8040868 can bind to these proteins NRSF, P300, USF1, and YY1 and also lead to Motifs changed. So we suspect that mutations in the rs8040868 (synonymous, Val53=) site would affect the targeted binding of the CHRNA3 gene to these proteins, which in turn affected the occurrence of LDH. The GTEx database has shown that rs8040868 mutation influences the expression of the *CHRNA3* in blood samples (https://gtexportal.org/home/). As briefly mentioned above, we cannot draw a firm conclusion on the biological effects of the SNPs on *CHRNA5*/*CHRNA3* cluster and future precise functional studies are worth considering.

## Conclusion

In conclusion, this study reported the potential association of genetic polymorphisms of *CHRNA5*/*CHRNA3* gene with LDH for the first time. Our results revealed a significant association of rs8040868 in *CHRNA3* with LDH, and *CHRNA5* haplotypes “TACACCCG” and “TACACCCG” are greatly related to the risk of LDH. Therefore, these findings may contribute to a better understanding of the pathogenic mechanisms of LDH and provide possible targets for treatment. Future studies should focus on the functional analysis and make the conclusion solid by replication in a similar study.

## Data Availability

All data and materials are available.

## Abbreviations

CIs: Confidence intervals
HWE: Hardy–Weinberg equilibrium
LD: Linkage disequilibrium
LDH: Lumbar disc herniation
MAF: Minor allele frequency
ORs: Odds ratios
SNPs: Single-nucleotide polymorphisms
